# miR-203 inhibits tumor cell migration and invasion via caveolin-1 in pancreatic cancer cells

**DOI:** 10.3892/ol.2014.1807

**Published:** 2014-01-16

**Authors:** LIFENG MIAO, XIANZE XIONG, YIXIN LIN, YAO CHENG, JIONG LU, JIE ZHANG, NANSHENG CHENG

**Affiliations:** Department of Bile Duct Surgery, West China Hospital, Sichuan University, Chengdu, Sichuan 610041, P.R. China

**Keywords:** pancreatic cancer, miR-203, caveolin-1

## Abstract

Pancreatic cancer is one of the most lethal malignant diseases with the poorest prognosis and is the fourth leading cause of tumor-associated mortality in the industrialized world. microRNAs (miRNAs or miRs) are small noncoding RNAs of approximately 22 nucleotides long that are able to function as oncogenes or tumor suppressors in human cancer. In our study, overexpression of miR-203 in Panc-1 cells is sufficient to reduce migratory ability and invasiveness, and to induce upregulation of epithelial markers (Snail, ZO-1 and β-catenin) followed by a decrease of mesenchymal marker expression (Zeb-1, vimentin and fibronectin). We also found that the caveolin-1 mRNA or protein levels are modulated by miR-203 in Panc-1 cells. We found that exogenous miR-203 altered the level of cell migration and invasion, and the expression of associated proteins following caveolin-1 knockdown by small interfering RNA. These results demonstrate that miR-203 inhibits cell migration and invasion via caveolin-1 in pancreatic cancer cells, suggest that miR-203 expression may be a useful indicator of the metastatic potential and provide a new therapeutic target in this common malignancy.

## Introduction

Pancreatic cancer is one of the most lethal malignant diseases with the poorest prognosis and is the fourth leading cause of tumor-associated mortality in the industrialized world (1). Due to the absence of specific symptoms and the lack of early detection, pancreatic cancer is usually diagnosed at an advanced and incurable stage. Therefore, the median overall survival is only 5–6 months after conventional therapies for locally advanced and metastatic disease, and the 5-year survival rate is only 5% (2–4). Such a short survival rate is primarily due to late diagnosis, intrinsic and extrinsic drug resistance, which contributes to tumor recurrence and metastasis. Epithelial to mesenchymal transition (EMT) represents a fundamental step in tumor invasion and metastasis (5). Tumor cells involved in EMT lose epithelial cell adhesion molecules and start to express mesenchymal-specific cytoskeletal components (6). This process is followed by morphological changes from a flattened epithelial to an elongated fibroblastic phenotype.

MicroRNAs (miRNAs or miRs) have important regulatory roles in controlling proliferation, development, differentiation and apoptosis through a fine modulation of key players in these cellular pathways (7). Several miR families have been implicated in EMT transition and EMT regulators differentially modulate their expression in healthy tissues compared to invasive tumors (8). miR-203 is a keratinocyte-derived miR that promotes epithelial differentiation from proliferative basal progenitors in the dermis by suppressing p63, a member of the p53 family (9,10). miR-203 is overexpressed in pancreatic adenocarcinoma compared with levels in normal pancreatic tissues and chronic pancreatitis, suggesting that miR-203 may be linked to specific characteristics of tumors and their progression patterns (11). miR-203 was also upregulated in the pancreatic cancer cells, as shown by miR array analysis, compared with normal human pancreatic duct epithelial cells, suggesting that miR-203 expression is a new prognostic marker in pancreatic adenocarcinoma patients (12). miR-203 transcription is specifically repressed by the EMT activator Zeb-1, contributing to the invasive and metastatic behavior of pancreatic and colorectal cancer cells (13).

In this study we show that miR-203 inhibits cancer cell migration, invasion and EMT transition, in pancreatic cancer. We also showed that miR-203 inhibits cell migration and invasion via caveolin-1. The present study suggests that miR-203 expression may be a useful indicator of the metastatic potential and provide a new therapeutic target in this common malignancy.

## Materials and methods

### Cell culture

The Panc-1 cell line, which was derived from an invasive intraductal extension of a primary tumor, had an intermediate expression level of caveolin-1 and was obtained from the Typical Culture Preservation Commission Cell Bank (Chinese Acadamy of Sciences, Shanghai. China). The stable human pancreatic cancer Panc-1 cell lines were cultured in DMEM medium (Invitrogen Life Technologies, Carlsbad, CA, USA) supplemented with 5% fetal bovine serum (FBS), 2 mmol/l glutamine, 50 units/ml penicillin and 50 μg/ml streptomycin (Invitrogen Life Technologies). The cells were maintained in a 5% CO_2_-humidified atmosphere at 37ºC.

### Transfection of miRNA mimics or small interfering RNA (siRNA)

Panc-1 cells were seeded at a density of 2×10^5^ cells per well in six-well plates and transfected with miR-203 mimics or negative miRNA control (GenePharma, Shanghai, China), or human caveolin-1 siRNA or a control siRNA (BIONEEC, Shanghai, China) using the using Lipofectamine 2000 (Invitrogen), following the manufacturer’s instructions. The media were removed after a 24-h transfection and the cells were incubated in media containing 5% FBS for an additional 24 h.

### Wound healing assay

A wound-healing assay was performed to examine the capacity for cell migration. Briefly, after the cells grew to 90% confluence in six-well plates, a single scratch wound was generated with a 200-μl disposable pipette tip. The scratch wounds were photographed over 7 h with a Nikon inverted microscope (Nikon, Tokyo, Japan) with an attached digital camera (DXM 1200, Nikon), and the scratch widths were quantitated with the ImageJ software (rsbweb.nih.gov/ij). The data were plotted as the percentage of wound closure, setting the initial scratch width as 100%.

### Cell invasion assay

The Transwell chambers (Millipore, Billerica, MA, USA) (8-μm pore size) were coated with Matrigel (BD Biosciences, Franklin Lakes, NJ, USA) (15 μg/filter). Cells (2.0×10^4^) in serum-free medium were plated into the upper chamber and the bottom wells were filled with complete medium. The cells were allowed to invade across the Matrigel-coated membrane for 72 h. Following incubation, the cells were removed from the upper surface of the filter by scraping with a cotton swab. The invaded cells that adhered to the bottom of the membrane were fixed with methanol and stained with DAPI. The number of cells that penetrated the membrane was determined by counting the mean cell number of five randomly selected high-power fields.

### Quantitative polymerase chain reaction (qPCR)

RNA was extracted using TRIzol (Invitrogen) or miRVANA (Ambion, Austin, TX, USA) kits according to the manufacturer’s instructions. Complementary DNA (cDNA) was generated with the High-Capacity cDNA Reverse Transcription kit (Roche Diagnostics GmbH, Mannheim, Germany). The quantitative analysis of the change in expression levels was calculated by the qPCR machine (iQ5, Bio-Rad, Hercules, CA, USA). qPCR was used to quantify the mRNA expression. The reactions were performed in duplicate and the Δ-Δ-cycle threshold values were calculated on the basis of the average of the normalization genes and the results were normalized to the average of the results obtained for glyceraldehyde 3-phosphate dehydrogenase (GAPDH) or RNU6B.

### Western blot analysis

The protein expression levels were assessed using western blot analysis. In brief, total cell lysates from different experiments were obtained by lysing the cells in RIPA buffer. The total cell lysates were separated on SDS-PAGE gels, transferred to PVDF membranes (Millipore), immunoblotted with antibodies [anti-snail rabbit polyclonal antibody, anti-ZO-1 rabbit polyclonal antibody, anti-β catenin rabbit polyclonal antibody, anti-vimentin rabbit polyclonal antibody, anti-fibronectin rabbit polyclonal antibody, anti-caveolin-1 rabbit polyclonal antibody, (all antibodies supplied by Proteintech, Wuhan, China), and anti-GAPDH monoclonal antibody (Bioworld, Nanjing, China)] and visualized using an enhanced chemiluminescence detection system (Amersham Biosciences, Chalfont St Giles, UK). The protein bands were quantitated by densitometry using gel analysis software ImageJ. The values were normalized to GAPDH expression.

### Statistical analysis

The results presented are the average of at least three experiments, each performed in triplicate with standard deviations. Statistical analyses were performed by analysis of variance followed by Tukey’s multiple comparison test or Student’s t-test using SPSS 15.0. P<0.05 was considered to indicate a statistically significant result and is indicated with asterisks.

## Results

### miR-203 inhibited cell migration and invasion in Panc-1 cells

**Figure 1:**
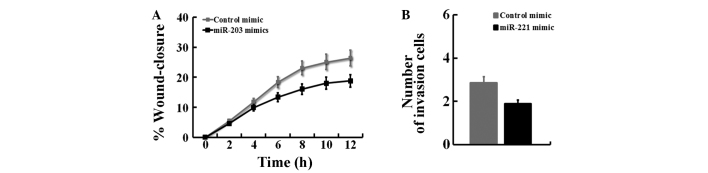
miR-203 mimics inhibit cell migration and invasion in Panc-1 cells. Panc-1 cells were treated with control or miR-203 mimics, then subjected to (A) the scratch wound assay and (B) cell invasion assays. The treatments in this figure were carried out in triplicate and the results are expressed as the means ± SD.

We first investigated whether miR-203 repressed cell migration using an *in vitro* scratch-wound assay. One day after the Panc-1 cells were treated with control or miR-203 mimics, a single scratch wound was created in the well, and the time-course of wound closure was monitored (Fig. 1A). In Panc-1 cells, treatment with miR-203 mimics strongly inhibited cell migration. To determine whether miR-203 affected the invasive ability of Panc-1 cells, we performed cell invasion assays using a Transwell system. The results of the cell invasion assay indicated that miR-203 inhibited the invasiveness of Panc-1 cells compared with blank cells, as indicated by a marked decrease in the number of cells that invaded the bottom well (P<0.05, Fig. 1B). These results suggest that miR-203 suppresses cell invasion in Panc-1 cells.

### miR-203 altered the expression of associated proteins in Panc-1 cells

**Figure 2:**
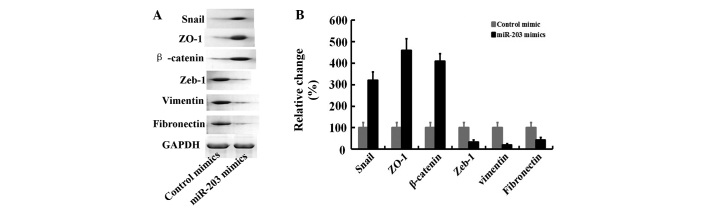
miR-203 altered the expression of associated proteins in Panc-1 cells. (A) Panc-1 cells were treated with control or miR-203 mimics, western blot analysis was used to determine the expression of associated proteins. (B) The relative proteins levels were normalized to GAPDH. The treatments in this figure were performed in triplicate, and the results are expressed as the means ± SD.

In this study, using western blot analysis, we also observed that miR-203 mimic treatment for 24 h significantly upregulated the expression of epithelial markers (Snail, ZO-1 and β-catenin) followed by a decrease of mesenchymal marker expression (Zeb-1, vimentin and fibronectin) (Fig. 2). These results suggest that miR-203 altered the expression of associated proteins of the EMT phenotype in Panc-1 cells.

### Regulation of caveolin-1 expression by miR-203 in Panc-1 cells

**Figure 3:**
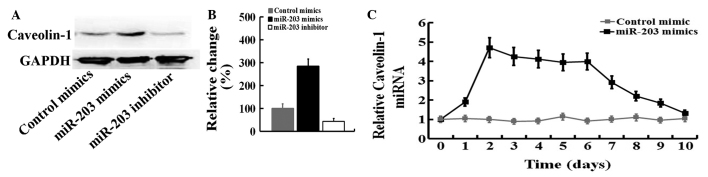
Caveolin-1 is regulated by the miR-203 in Panc-1 cells. (A) The caveolin-1 protein levels in Panc-1 cells treated with control or miR-203 mimics, or miR-203 inhibitor. (B) The relative proteins levels were normalized to GAPDH. (C) Panc-1 cells were treated with control or miR-203 mimics, and subjected to quantitative polymerase chain reaction of caveolin-1. The treatments in this figure were performed in triplicate and the results are expressed as the means ± SD. GAPDH, glyceraldehyde 3-phosphate dehydrogenase.

We investigated whether the caveolin-1 mRNA or protein levels are modulated by miR-203 in Panc-1 cells. We observed that the caveolin-1 protein level was increased by approximately 330% (Fig. 3A and B) upon treatment of Panc-1 cells with miR-203 for 24 h under conditions that significantly inhibited the expression of mesenchymal markers (Fig. 2). By contrast, miR-203 inhibitor was able to inhibit caveolin-1 protein expression. A time-course of the expression of caveolin-1 was examined after miR-203 mimics-treatment in Panc-1 cells. Caveolin-1 mRNA was induced 4.7-fold after 2 days of treatment with miR-203 mimics and gradually decreased after 7 days (Fig. 3C), suggesting that caveolin-1 is likely to be regulated by miR-203 signaling.

### Downregulation of caveolin-1 expression promoted cell migration and invasion in Panc-1 cells

**Figure 4:**
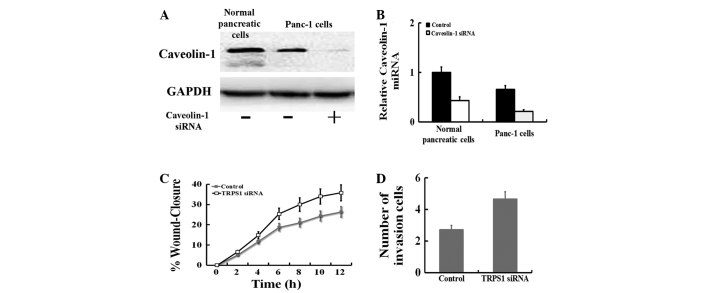
Downregulation of caveolin-1 expression promoted cell migration and invasion in Panc-1 cells. (A) Western blot analysis and (B) quantitative polymerase chain reaction were used to determine the protein levels of caveolin-1 in normal pancreatic cells and Panc-1 cells transfected with vehicle or caveolin-1 siRNA. Panc-1 cells were treated with control or caveolin-1 siRNA, then subjected to (C) the scratch wound assay and (D) cell invasion assays. The treatments in this figure were performed in triplicate and the results are displayed as the mean ± standard deviation. GAPDH, glyceraldehyde 3-phosphate dehydrogenase.

The baseline mRNA and protein levels of caveolin-1 in Panc-1 cells and normal pancreatic cells were determined by qPCR and western blotting. The results showed that the mRNA and protein of caveolin-1 was highly expressed in Panc-1 cells compared with normal pancreatic cells (P<0.05; Fig. 4A). Thus, Panc-1 cells were transiently transfected with caveolin-1 siRNA. Compared with the blank (no siRNA) Panc-1 cells, the expression of FoxM1 was markedly suppressed in cells transfected with caveolin-1 siRNA at the mRNA (P<0.05) and protein levels. In this study, we also observed that caveolin-1 siRNA treatment significantly promoted cell migration and invasion in Panc-1 cells (Fig. 4C and D).

### miR-203 inhibits the EMT by targeting caveolin-1

**Figure 5:**
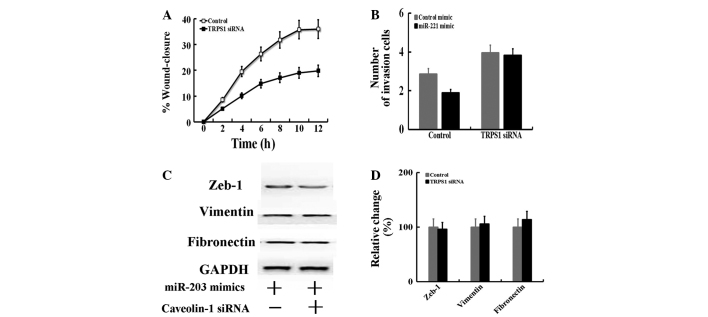
miR-203 inhibits the EMT by targeting caveolin-1. Panc-1 cells were transfected with control or caveolin-1 siRNA. The cells were then treated with the miR-203 mimic for 1 day and subjected to (A) the scratch-wound assay, (B) cell invasion assays and (C) western blot analysis of Zeb-1, vimentin and fibronectin. (D) The relative proteins levels were normalized to GAPDH. The treatments in this figure were carried out in triplicate and the results are displayed as the means ± SD. GAPDH, glyceraldehyde 3-phosphate dehydrogenase; EMT, epithelial to mesenchymal transition.

To assess whether caveolin-1 is responsible for the miR-203-dependent inhibition of cell migration and invasion, the miR-203 mimic was transfected into Panc-1 cells treated with caveolin-1 siRNA, and then to examine whether induction of caveolin-1 by miR-203 plays a role in cell migration (Fig. 5A) and invasion (Fig. 5B). When caveolin-1 was silenced by caveolin-1 siRNA, miR-203 did not suppress cell migration and invasion. By contrast, transfection of the miR-203 mimic alone significantly elevated cell migration (Fig. 1A) and invasion (Fig. 5B). The protein of Zeb-1, fibronectin and vimentin were examined by western blot analysis (Fig. 5C). We observed that exogenous miR-203 altered the expression of associated proteins following caveolin-1 knockdown by siRNA. These results indicate that caveolin-1 is essential for miR-203-dependent cell migration and invasion.

## Discussion

miR-203 is an antiproliferative microRNA involved in skin differentiation that targets the 3′-UTR of the ‘stemness-maintaining’ transcription factor ΔNp63α (14). The downregulation of miR-203 expression has been described in several types of cancer, including hepatocellular carcinomas (15), breast cancer (16) and pancreatic cancer (12). miR-203 acts as a tumor suppressor in chronic myelogenous leukemias, acute lymphoblastic leukemias and in hepatocellular carcinomas where its expression is silenced by chromosomal deletion or promoter CpG island hypermethylation (17). miR-203 transcription is specifically repressed by the EMT activator Zeb-1, contributing to the invasive and metastatic behavior of pancreatic and colorectal cancer cells (16).

A previous study showed that miR-203 is downregulated in metastatic pancreatic cancer cell lines compared to normal epithelial pancreatic cells, a result that suggests that miR-203 deficiency contributes to prostate cancer progression and metastasis (12). Previous results have correlated miR-203 downregulation with increased proliferative, invasive and metastatic potential of transformed cells in other malignancies (14). In the present study, we showed that this is also true in pancreatic cancers and, using the metastatic cell line (Panc-1), we investigated the molecular mechanisms downstream of miR-203. *In vitro* wound healing assays and Transwell/Matrigel invasion assays following miR-203 transfection in Panc-1 cells suggest that its expression is sufficient to reduce migratory ability and invasiveness. Overexpression of miR-203 in Panc-1 cells was sufficient to induce upregulation of epithelial markers (Snail, ZO-1 and β-catenin) and was followed by a decrease of mesenchymal marker expression (Zeb-1, vimentin and fibronectin).

In our study, we have identified caveolin-1 as miR-203 direct target mRNAs involved in these events. We observed that the caveolin-1 mRNA or protein levels are modulated by miR-203 in Panc-1 cells. Previous studies indicated that caveolin-1 is a crucial modulator of EMT and cell differentiation in pancreatic cancer cells (18). Using transfected with caveolin-1 siRNA suppress the expression in the Panc-1 cells that showed the least caveolin-1 expression. The absence of caveolin-1 expression was sufficient to promote pancreatic cancer cell migration and invasion. When caveolin-1 was silenced by caveolin-1 siRNA, miR-203 did not alter the level of cell migration and invasion. We observed that exogenous miR-203 altered the expression of associated proteins after caveolin-1 knockdown by siRNA. Our data suggest that miR-203 inhibits cell migration and invasion via caveolin-1 in pancreatic cancer cells.

miR-203 pleiotropically regulates the expression of a cohort of effectors involved in cancer stem cell self-renewal, cytoskeletal remodeling and tissue-specific metastatic spreading pathways. Our studies suggest that miR-203 may also be considered a suppressor of cancer cell migration, invasion and EMT transition in pancreatic cancer, and that miR-203 expression may be a useful indicator of the metastatic potential and provide a new therapeutic target in this common malignancy.
